# High Diversity of β-Glucosidase-Producing Bacteria and Their Genes Associated with Scleractinian Corals

**DOI:** 10.3390/ijms22073523

**Published:** 2021-03-29

**Authors:** Hongfei Su, Zhenlun Xiao, Kefu Yu, Qi Zhang, Chunrong Lu, Guanghua Wang, Yinghui Wang, Jiayuan Liang, Wen Huang, Xueyong Huang, Fen Wei

**Affiliations:** 1 Coral Reef Research Center of China, Guangxi Laboratory on the Study of Coral Reefs in the South China Sea, School of Marine Sciences, Guangxi University, Nanning 530004, China; shf2016@gxu.edu.cn (H.S.); xzlgxu@sina.com (Z.X.); zhangqi1780@163.com (Q.Z.); chunrlu@163.com (C.L.); wgh@gxu.edu.cn (G.W.); wyh@gxu.edu.cn (Y.W.); jyliang@gxu.edu.cn (J.L.); wenhuang@gxu.edu.cn (W.H.); huangxueyong@gxu.edu.cn (X.H.); weifensky@163.com (F.W.); 2 Southern Marine Science and Engineering Guangdong Laboratory, Zhuhai 519080, China

**Keywords:** scleractinian corals, diversity, cultivable bacteria, β-glucosidase, metagenomic approach

## Abstract

β-Glucosidase is a microbial cellulose multienzyme that plays an important role in the regulation of the entire cellulose hydrolysis process, which is the rate-limiting step in bacterial carbon cycling in marine environments. Despite its importance in coral reefs, the diversity of β-glucosidase-producing bacteria, their genes, and enzymatic characteristics are poorly understood. In this study, 87 β-glucosidase-producing cultivable bacteria were screened from 6 genera of corals. The isolates were assigned to 21 genera, distributed among three groups: Proteobacteria, Firmicutes, and Actinobacteria. In addition, metagenomics was used to explore the genetic diversity of bacterial β-glucosidase enzymes associated with scleractinian corals, which revealed that these enzymes mainly belong to the glycosidase hydrolase family 3 (GH3). Finally, a novel recombinant β-glucosidase, referred to as Mg9373, encompassing 670 amino acids and a molecular mass of 75.2 kDa, was classified as a member of the GH3 family and successfully expressed and characterized. Mg9373 exhibited excellent tolerance to ethanol, NaCl, and glucose. Collectively, these results suggest that the diversity of β-glucosidase-producing bacteria and genes associated with scleractinian corals is high and novel, indicating great potential for applications in the food industry and agriculture.

## 1. Introduction

Reef-building corals are highly subtle and complex holobionts that consist of the cnidarian host and its associated microbes, including symbiodiniaceae, protists, fungi, archaea, bacteria, and viruses [1,2]. Coral-associated bacteria play fundamental roles in biological processes and the overall fitness of their environment, and are both abundant and diverse [3]. Previous studies estimated the number of prokaryotic cells from direct in situ cell counts of coral mucus and coral tissue to be 1 × 10^6^ cells/mL [4] and 10^7^ cells/cm^2^ [5], respectively. Early studies of coral microbiology, using culture-free and 16S rRNA sequencing-DNA-based techniques, were first applied to the study of coral associated bacteria [6], enabling investigators to identify a wider range of bacterial groups [7]. With the development of high-throughput sequencing technology, and a corresponding decrease in sequencing costs, many bacterial phyla, including some candidate phyla, have been identified as being associated with corals. Previous studies have shown that dominant bacterial phyla, with an abundance of over 90%, are similar among different coral species, for example, Proteobacteria, Bacteroidetes, Firmicutes, Cyanobacteria, and Chloroflexi [8,9]. In addition, the coral core microbiome, which includes rare bacterial taxa, has been identified as ubiquitous endosymbionts [10]. Many studies support the notion that the microbiome of corals is distinct from that of the overlying seawater, and that bacterial communities supported by corals fluctuate with the season [9,11] and are host-specific [12,13], geographically consistent [6], and physiologic [14,15]. Nevertheless, the number of bacterial strains isolated from coral is very small [9,16], and their specific physiological roles have not been fully elucidated.

As an integral part of the coral holobiont, bacteria provide important nutrients and other resources, including nitrogen and carbon, to the cnidarian host [17]. For example, dissolved organic carbon (DOC) is accumulated through carbon fixation via photosynthesis and consumed by heterotrophic bacteria, which has been confirmed in sediment samples where organic-carbon-killing of corals, ranging from simple sugars, polysaccharides, and complex compounds, is negated by antibiotics [18,19]. Metagenomic analysis of these coral-associated microbes shows that a diverse array of enzymes capable of carbon fixation and carbon degradation are present and capable of metabolizing coral mucus and transporting the resulting sugars into their cells for energy [20,21]. A series of genes implicated in the degradation of simple sugars and/or complex carbohydrates, such as cellulose through an array of cellulases, were also identified [21]. Moreover, plankton allows corals to replenish sufficient carbon to meet their metabolic energy requirements, leading to reduced mortality from bleaching. This is likely because fixed carbon is heterotrophically acquired during recovery from bleaching, and before photoautotrophic acquisition resume [22]. In addition, corals may digest zooxanthellae, whose cell walls are composed of a stable shell of cellulose to establish a better symbiotic relationships [23]. However, coral hosts such as *Galaxea fascicularis* may be deficient in cellulase activity, and thus, such enzymatic activity could be attributed to bacteria surrounding the corals [24]. More than 10 glycosidases from commensal bacteria of *Photobacterium mandapamensis* were modestly induced on coral mucus, involving the regulation of galactose, glucose, arabinose, mannose and N-acetyl-glucosamine, and related to coral host defense [25]. These studies imply that bacterial cellulases may play a key role in regulating energy and providing defense against free radicals in coral. To date, there is virtually no information on the bacterial species and the diversity of cellulase-producing bacteria isolated from coral.

Among the three types of bacterial cellulases (EC 3.2.1.21) in glycoside hydrolase family of enzymes, β-glucosidases represent an important and ubiquitous class of hydrolases because they are capable of cleaving polysaccharides, the most abundant class of chemically identifiable compounds in the oceanic dissolved organic matter (DOM) pool, to available monosaccharide for consumption by biological organisms [26,27,28]. The diversity of β-glycosidase-producing bacteria has been studied in different habitats, including soil [29], cow dung and compost [30], and marine environments. The first study investigating the characteristics and diversity of β-glucosidase activity was reported in marine snow, where a β-glucosidase isoenzyme was detected by two different chromatographic separations and the same isoenzyme exhibited two peak of activity when eluted at lower NaCl concentrations [31]. Subsequently, Arrieta et al. proposed a novel method using capillary electrophoresis zymography for the rapid separation and detection of marine bacterial β-glucosidases, providing evidence of significant diversity in the coastal North Sea [32]. A further study linked function and diversity of the microbial β-glucosidase to microbial community structure [33,34] and coastal phytoplankton blooms [35]. However, there are no reports focused on bacterial β-glucosidase enzymes from corals, which may have significant physiological effects on corals and their symbionts.

Although being considered “oligotrophic areas,” coral reefs are among the most biologically diverse and productive ecosystems on the planet [36], within which symbiotic microbes grow up to 50 times faster than in open ocean communities that processes fatally driving organic carbon flow [37]. A diverse array of bacteria capable of the degradation of simple sugars and complex carbohydrates, such as cellulases and chitinases, have been identified [21]. Only two representative coral commensals bacteria, including *Photobacterium mandapamensis* and *Halomonas meridiana*, and a white pox pathogen *Serratia marcescens*, have been isolated and characterized with respect to their glycosidase activity [25]. However, both the diversity and essential characteristics of β-glycosidase-producing organisms have been poorly understood. In this study, β-glycosidase-producing microorganisms were isolated from six genera of corals, and their diversity was investigated via a phylogenetic analysis of their 16S rDNA gene sequences. The diversity of bacterial β-glycosidase genes was studied using a metagenomic approach. A novel gene, Mg9373, was isolated from coral microorganisms by high-throughput sequencing and functional screening. The recombinant enzyme was cloned and expressed in *Escherichia coli* BL21(DE3) and its enzymatic properties were characterized. This study aimed to explore the diversity of cultivable β-glycosidase-producing bacterial communities and their extracellular β-glycosidases associated with scleractinian corals.

## 2. Results

### 2.1. Diversity of β-Glucosidase-Producing Bacteria Associated with Scleractinian Corals

**Figure 1:**
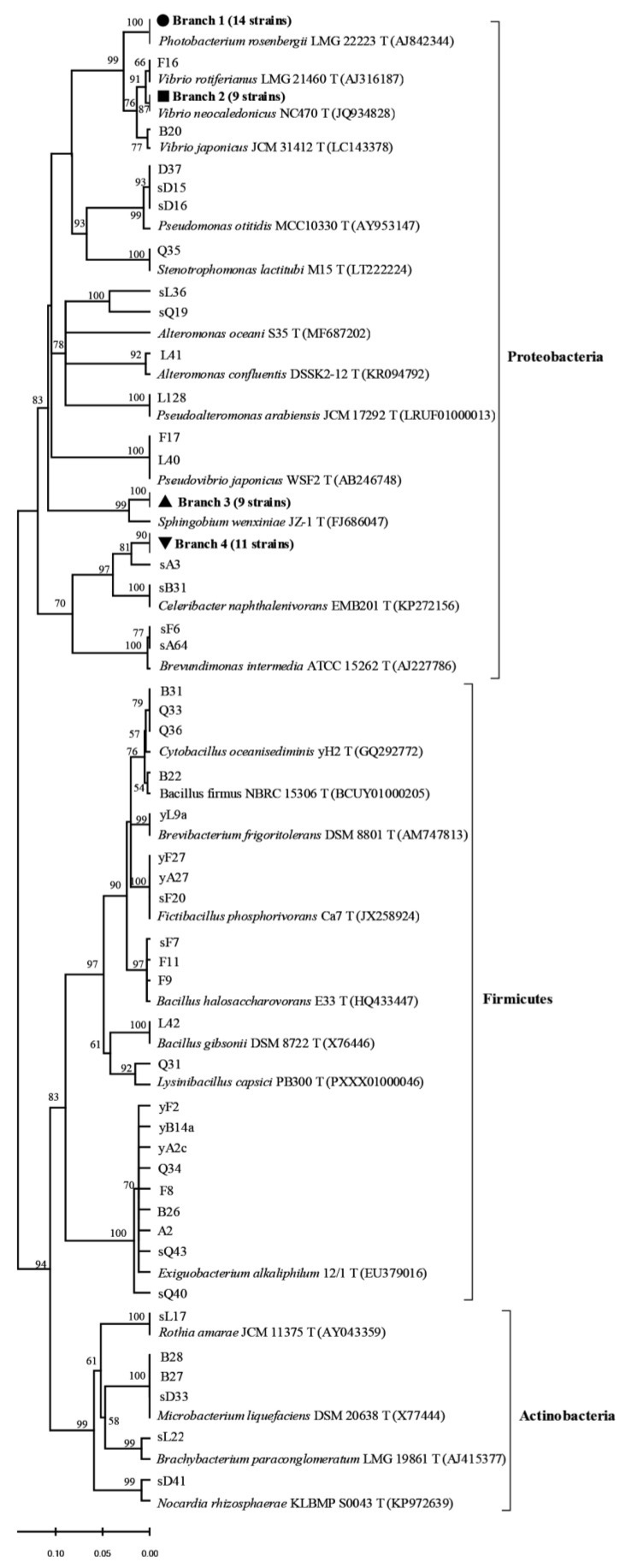
Phylogenetic tree of the cultivable β-glucosidase-producing bacteria isolated from scleractinian corals based on 16S rRNA gene sequences. The tree was constructed by the neighbor-joining method using MEGA package version 5.0. Only bootstrap values greater than 50% are presented in the nodes. The scale bar represents 2% nucleotide substitution. Branch 1 indicates nine *Vibrio* strains similar to *Vibrio rotiferianus* LMG21460T (AJ316187). Branch 2 indicates 13 Photobacterium strains similar to *Photobacterium rosenbergii* LMG 22223 T (AJ842344). Branch 3 indicates eight *Sphingobium* strains similar to *Sphingobium wenxiniae* JZ-1 T (FJ686047). Branch 4 indicates 11 *Brevundimonas* strains similar to *Brevundimonas intermedia* ATCC 15262 T (AJ227786). The circle indicated that 14 strains were from *Favia*, and the sequence similarity between them and *Photobacterium rosenbergii* was 99%, which were clustered into branch 1; The square indicates that a total of 9 strains isolated from *Pocillopora*, *Porites*, *Acropora* and *Montipora* have sequence similarity of 98.5–99.2% with *Vibrio rotierianus*, and clustered into branch 2; The trigonometry indicates that 9 strains with 97.5–99.1% similarity to *Sphingobium wenxiniae* JZ-1 clustered into branch 3; The inverted triangle indicated that the 11 strains were most similar to *Paracoccus marcusii* in similarity and clustered into branch 4. *Pocillopora*, *Acropora*, *Porites*, *Favia*, *Turbinaria,* and *Montipora* are represented by A, B, C, D, E, and F.

A total of 87 coral symbiotic β-glucosidase-producing bacteria were identified based on 16S rRNA gene sequences and belonging to 21 genera. Most bacteria were classified into Firmicutes and Proteobacteria, except for three isolates (b27, b28 and sD33) belonging to *Microbacterium* from *Turbinaria peltate*, and *Rothia* (sL17), *Brachybacterium* (sL22), and *Nocardia* (sD41) belonging to Actinobacteria. The genera include *Vibrio* (11 strains), *Alteromonas* (3 strains), *Stenotrophomonas* (1 strains), *Pseudomonas* (3 strains), *Sphingobium* (9 strains), and *Brevundimonas* (2 strains) in Proteobacteria (59 strains) and *Exiguobacterium* (9 strains), *Bacillus* (5 strains), *Fictibacillus* (3 strains)*, Brevibacterium* (1 strain), *Lysinibacillus* (1 strain), and *Cytobacillus* (3 strains) in Firmicutes (22 strains). *Photobacterium* (28.57%), *Paracoccus* (22.49%) and *Vibrio* (22.45%) were the predominant genera in Proteobacteria, *Exiguobacterium* (40.91%) was the most dominant in Firmicutes, and *Lysinibacillus* (Q31), *Stenotrophomonas* (Q35), *Celeribacter* (sB31), *Brevibacterium* (yL9A), *Bacillus* (L42), *Rothia* (sL17), *Nocardioides* (sD41), and *Brachybacterium* (sL22) were represented by only one isolate each (eight isolates), corresponding to 9.19% of the total isolates (Figure 1 and Table 1). *Exiguobacterium* and *Vibrio* were isolated in five genera of coral, but found to be most dominant in *Montipora* and *Porites* (Table 1). *Photobacterium* and *Paracoccus* were only found in *Favia*, *Pocillopora*, and *Porite* (Table 1). Furthermore, the β-glucosidase-producing bacteria identified from *Porites*, *Favia*, and *Acropora* belonged to eight genera, exhibiting higher diversity than those isolated from other corals. Only four genera were isolated from *Turbinaria*, which represented the least diverse community among all of the coral samples (Table 1).

A neighbor-joining phylogenetic tree was constructed, including all related sequences from this study and reference sequences from the GenBank database (Figure 1). Approximately 14 strains from *Faviia* formed Branch 1, as shown in Figure 1, revealing 99% sequence similarity with *Photobacterium rosenbergii* strain. Out of the 11 *Vibrio* isolates, 9 isolates from *Pocillopora*, *Porites*, *Acropora,* and *Montipora* formed Branch 2 (Figure 1), revealing 98.5–99.2% sequence similarity with *Vibrio rotierianus* LMG21460T, which was isolated from the gut of Pacific white shrimp and the rearing waters of Malaysia and Vietnam. Nine *Sphingobium* strains (Branch 3 from three corals) clustered with *Sphingobium wenxiniae* JZ-1, revealing 97.5–99.1% similarity. Eleven (Branch 4 from four corals) were closest to *Paracoccus marcusii*. The phylogenetic relationships of the other strains are shown in Figure 1. These results suggest that β-glucosidase-producing bacterial diversity and composition varied among the different genera of coral, and the highest diversity and evenness in the community were present in *Porites* and *Acropora* (Table 1).

### 2.2. Diversity of the β-Glucosidase Gene

**Figure 2:**
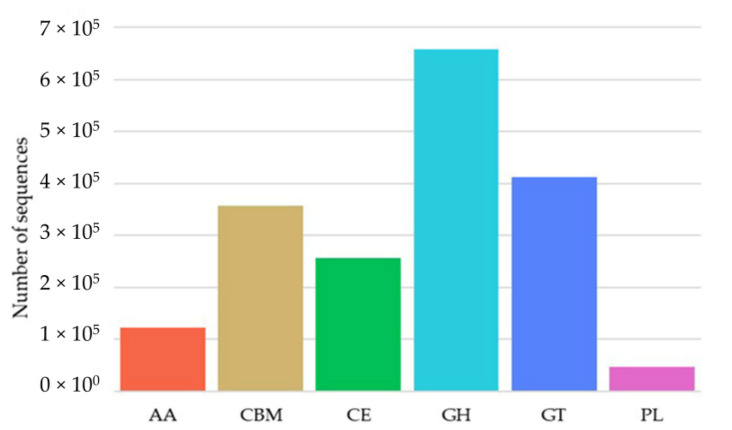
Carbohydrate-active enzymes: Auxiliary Activities (AA); Carbohydrate-Binding Modules (CBM); Carbohydrate Esterases (CE); Glycoside Hydrolases (GH); Glycosyl Transferases (GT); Polysaccharide Lyases (PL).

**Figure 3:**
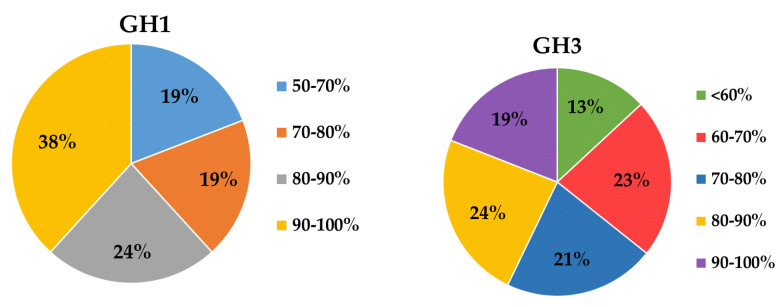
Amino acid sequence homologies of GH1 and GH3 β-glucosidase gene fragments from coral microbiology metagenomic DNA to known β-glucosidase.

**Figure 4:**
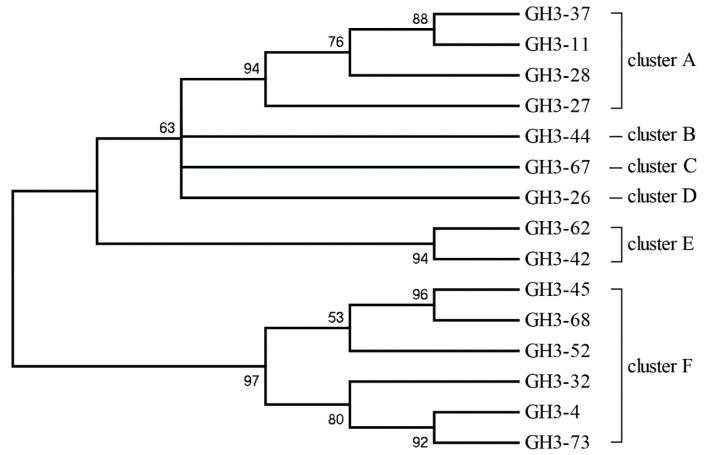
Maximum Likelihood phylogenetic tree of the GH3 amino acid sequences. Amino acid sequences larger than 500 were selected from the GH3 family with the largest number for tree building analysis.

The genomic DNA of 87 cultivable β-glucosidase-producing bacteria was purified and used to construct a clone library. After DNA high-throughput sequencing and protein and gene ontology annotation, out of 1,853,698 sequences identified as being related to carbohydrate metabolism and energy, 658,202 sequences were identified as glycoside hydrolase (GH) gene fragments (Figure 2). A total of 68 and 84 sequences were identified as GH1 and GH3 glucosidase gene fragments, respectively, based on BLASTx analysis, after the removal of redundant sequences (Table 2). The lowest genetic similarities were observed to have 37% homology with β-glucosidase data from Gene Bank, while some sequences showed sequence homology values of up to 100%, for example the β-glucosidase from *Bacillus thuringiensis* strain GA-A07. Moreover, almost 60% of sequences showed divergence, and had low levels of similarity (80%) with known β-glucosidase sequences in GenBank (Figure 3), implying that there may be an abundance of novel β-glucosidase enzymes in coral symbiotic microorganisms. An unrooted protein-level phylogenetic tree of GH3 β-glucosidase was constructed using 15 divergent sequences that contain more than 500 amino acids. All sequences were confined to six clusters, denoted as Clusters A to F, indicating substantial diversity among the GH3 family of β-glucosidases in coral symbiotic bacteria (Figure 4). The presence of clades without close relatives suggests their novelty, which might be due to a large portion of novel genes in coral bacteria.

### 2.3. Expression and Purification of Mg9373

**Figure 5:**
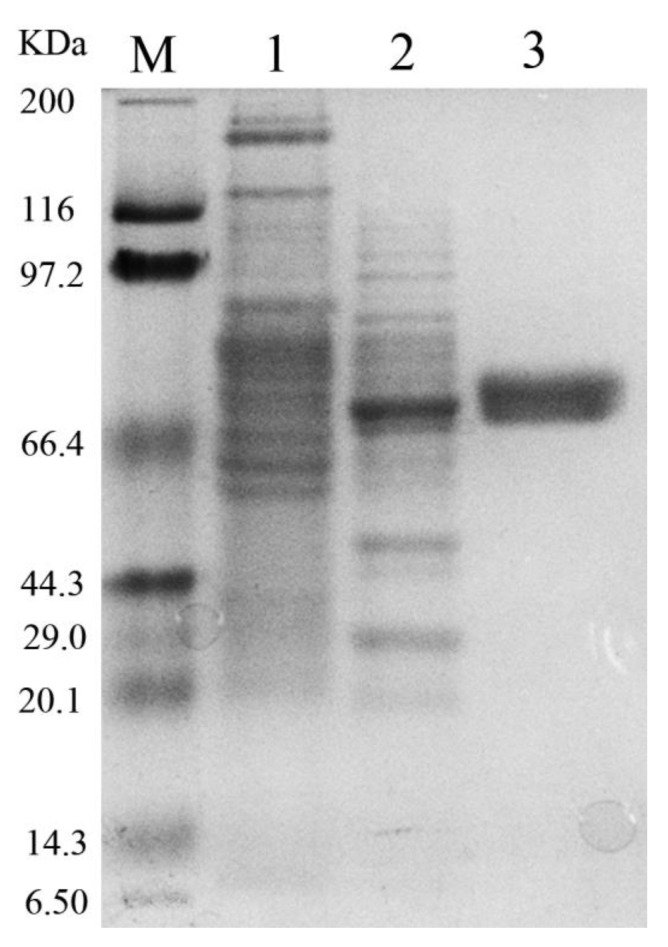
Molecular weight of Mg9373 determined by SDS-PAGE. M: protein molecular weight markers; 1: supernatant of cell lysis from E. coli BL21 DE3 cells; 2: supernatant of cell lysis from recombinant E. coli BL21 DE3 cells harboring pEASY-E1-mg9373 plasmid; 3: purified enzyme Mg9373 from Ni^2+^ column.

A fragment sequence denoted mg9373 was selected to obtain the full-length gene, because it was the most abundant of all the sequences, and represents a novel enzyme having low homology (70%) with known β-glucosidases. The complete sequence of Mg9373 contained an open reading frame of 1902 bp encoding a 633 amino acid polypeptide, without a typical signal peptide. The recombinant Mg9373 protein, which contains an N-terminal 6×His-tag, was purified using Ni^2+^-NTA chromatography. SDS-PAGE analysis showed that Mg9373 was successfully purified to homogeneity, with a molecular mass approximately of 75.0 kDa, which corresponds to the calculated mass (Figure 5).

### 2.4. Biochemical Characterization of Mg9373

**Figure 6:**
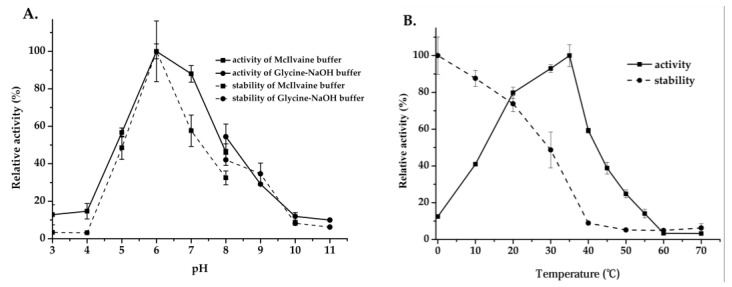
Effect of pH and temperature of Mg9373 on active and stability. (**A**) Effect of pH on active. The effect of pH on Mg9373 activity was determined at McIlvaine buffer between pH 3.0–8.0 and Glycine-NaOH buffer between pH 8.0–11.0, at 40 °C. (**B**) ffect of temperature on active and thermostability assay. The activity was determined in McIlvaine buffer (pH 7.0) at various temperatures (0–70 °C) and the thermostability of Mg9373 was determined after incubation at various temperatures (0–70 °C).

**Figure 7:**
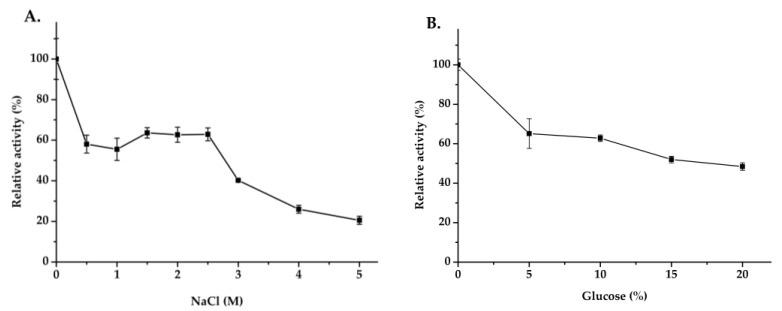
Effect of NaCl (**A**) and gluose (**B**) of Mg9373 on active and stability. (**A**) Effect of NaCl on stability. The stability of Mg9373 was determined at 40 °C in McIlvaine buffer (pH 6.0) after incubation at various NaCl (0–5 M). (**B**) Effect of glucose stability. The stability of Mg9373 was determined after incubation at various glucose (0–20%), at 40 °C in McIlvaine buffer (pH 6.0).

**Figure 8:**
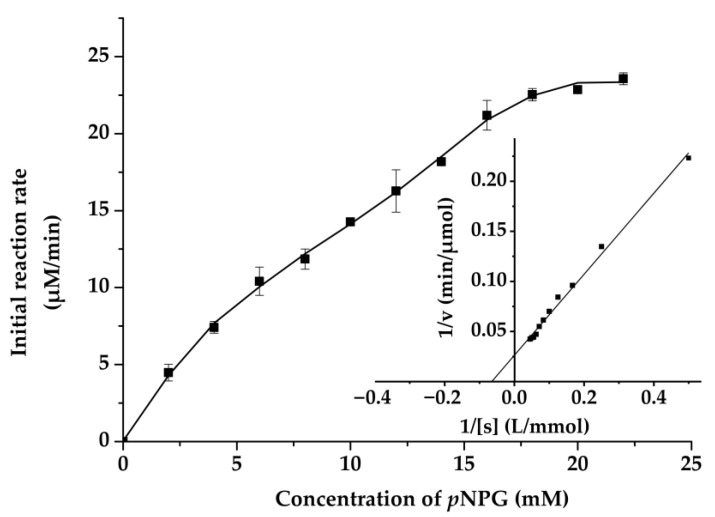
Michaelis-Menten plots for the reactions with pNPG. The inset shows the Lineweaver-Burk plots. Each data point represents the mean ± SD of three independent experiments.

The effect of pH and temperature on enzyme activity and stability are shown in Figure 6A,B, and the optimal pH and temperature of Mg9373 were found to be 6.0 and 35 °C, respectively. The enzyme retained >80% of the maximal activity at pH 7, and exhibited a rapid drop, while pH values remained above 7.0 and below 6.0 (Figure 6A). The activity of Mg9373 remained at a high level (>40%) when the temperature was maintained between 10 °C and 45 °C (Figure 6B), indicating that the enzyme is amenable to a wide range of temperature conditions. The effects of metal ions and other additives on the activity of Mg9373 is summarized in Table 1. We found that Ni^+^, Zn^2+^, Mn^2+^, Mg^2+^, Co^2+^, Ca^2+^, K^+^, and EDTA significantly increased enzyme activity in a short period of time. Most metal ions, at a concentration of 1 mM, enhanced Mg9373 activity, with no significant inhibition to activity observed at concentrations up to 20 mM, with the exception of Zn^2+^, Cu ^2+^, and Hg^2+^. Specifically, the activity increased more than three-fold in 10 mM Mn^2+^, and 1.4-fold in 10 mM of Zn^2+^; however, enzymatic activity decreased to 76% in 20 mM Zn^2+^ (Table 3). In general, the activity of enzymes is also enhanced by the addition of organic reagents (Table 2); however, most organic reagents were found to inhibit the activity of Mg9373. Specifically, the enzymatic activity of Mg9373 increased to 148%, 153%, and 115% in the presence of 20% benzene, 20% toluene, and 10% hexane, respectively (Table 4) Interestingly, Mg9373 was tolerant to ethanol with 57.8% relative activity at 10% ethanol (*v*/*v*), though sensitive to methanol, acetonitrile, and butanol. Mg9373 displayed good tolerance to salinity, as 60% and 40% of its relative activity remained in the presence of 2.5 M and 3 M NaCl, respectively (Figure 7A). In addition, Mg9373 demonstrated a high tolerance to glucose, with activity levels of 65.2%, 62.8%, 51.9%, and 48.4% at 5%, 10%, 15%, and 20% glucose, respectively (Figure 7B). Lastly, a kinetic analysis of Mg9373 revealed a *K*_m_ of 15.11 mM, and a *V*_max_ value of 37.467 μM·L^−1^·min^−1^ (Figure 8) toward *p*NPG. 

## 3. Discussion

This study presents the first attempt to examine the diversity of β-glucosidase-producing bacteria associated with scleractinian corals. Eighty-seven β-glucosidase-producing cultivable bacteria were screened from six genera of corals. All isolates were assigned to 21 genera, which were distributed among Proteobacteria, Firmicutes, and Actinobacteria. Among of them, Firmicutes and Proteobacteria accounted for the majority of β-glucosidase-producing bacteria, except for six isolates (b27, b28, sD33, sL17, sL22, and sD41) belonging to Actinobacteria. The metagenomic analysis revealed that the β-glucosidase enzymes identified mainly belonged to the GH3 family, which were divided into six clusters. A novel recombinant β-glucosidase, namely Mg9373, which consisted of a total of 670 amino acids and was classified as a member of the GH3 family, exhibited excellent tolerance to ethanol, NaCl, and glucose. 

### 3.1. Diversity Analysis of β-Glucosidase-Producing Bacteria Associated with Scleractinian Corals

It should be noted that less than 1% of microorganisms in coral can be cultured in viable count procedures, though their actual number is several orders of magnitude higher. However, abundant and diverse coral-associated bacteria play fundamental roles in physiological processes and the overall fitness of their marine environments [3]. It has been confirmed that DOC accumulation is due to bacteria, with carbon sources ranging from simple sugars to polysaccharides [18,19]. Diverse microbial populations in herbivore rumen enables the digestion of complex cellulose materials into more absorbable nutrients, where conditions parallel to this may be associated with the coral polyp [38]. On the one hand, coral symbiotic bacterial enzymes are able to digest active predation to replenish sufficient carbon for meeting animal host metabolic energy requirements even when photoautotrophic acquisition resumed [22], resulting in less mortality from bleaching than poor plankton consumers. Among them, β-glucosidases are one of most ubiquitous hydrolases capable of cleaving polysaccharides into monosaccharides, which are more easily absorbed by living organisms [26,27,28]. Corals may also digest zooxanthellae, which have cell walls composed of a stable shell of cellulose, to establish a better symbiotic relationship [23]. However, coral hosts, such as *Galaxea fascicularis*, may be deficient in cellulase activity, which could be supplemented by symbiotic bacteria [24]. More than 10 glycosidases in the commensal bacteria of *P. mandapamensis* were modestly induced during incubation on coral mucus that were object to regulate galactose, glucose, arabinose, mannose, and N-acetyl-glucosamine, which related to coral host defense [25]. Previous studies in related fields have demonstrated that the activity and diversity of β-glucosidases are significantly driven by shifts in the bacterial community structure, rather than by simple induction of enzyme expression during coastal phytoplankton blooms [35] and soil aggregation [39]. Culture-dependent methods have revealed the presence of numerous extracellular enzyme-producing microorganisms in corals [16]. In our study, 87 cultivable β-glucosidase-producing bacteria obtained from coral were divided into 17 genera and classified into Proteobacteria and Firmicutes, except for four isolates belonging to Actinobacteria. In comparison with the soil study, where the primary β-glucosidase-producing microorganisms identified were classified as *Sphingomonas* sp. (22%), *Burkholderia* sp. (20%), *Luteibacter* sp. (15%), and *Streptomyces* sp. (15%), we found that *Photobacterium* (28.57%), *Paracoccus* (22.49%), and *Vibrio* (22.45%) were the predominant genera in coral. Moreover, the β-glucosidase-producing bacteria identified from *Porites*, *Favia*, and *Acropora* exhibited higher diversity than those isolated from other corals. *Acropora* sp. generally grow faster than other corals and have been hypothesized to be more sensitive to ocean warming and acidification [40]. A possible explanation could be that massive corals, such as *Porites* and *Favia*, have a higher tolerance to environmental stress because of the diversity and complexity of the associated bacterial community, which provides them with higher energy reserves and photo-protective capacities [13,15,41]. Taken together, these results indicate that certain β-glucosidase-producing bacteria are indeed specific to corals, where they play an important role in their host response to changes in the immediate environment and through periods of severe crisis of bleach due to heterotrophy to meet their polyp metabolic energy requirements. 

### 3.2. Diversity Analysis of β-Glucosidase Gene

Protein and gene ontology annotation revealed that glycoside hydrolase (GH) gene fragments represent the main gene pool related to carbohydrate metabolism and energy production, and were identified in all 87 β-glucosidase-producing bacteria. In total, 68 GH1 and 84 GH3 glucosidase gene fragments were identified. These findings differ from those identified in an analysis of the hindgut of *Holotrichia parallela larvae*, which can degrade pure cellulose or neutral detergent fibers by their intestinal bacteria and its gut may be a potential source of glycosyl hydrolase, in which the main families were GH2, GH8, GH10, and GH36 [42]. Approximately 46% (38/84) of the sequences exhibited low similarity (70%) with known β-glucosidase sequences in the GenBank (Figure 4), implying that they may be novel β-glucosidases. This is much higher than that found in the rumen metagenome of cows [43], rabbits [44], and buffalos [45], in a previous investigation. 

### 3.3. Expression and Purification of Mg9373

Sequence similarity searches revealed that Mg9373 shared the highest homology (70%) with 1,4-beta-glucosidase from *Vibrio harveyi* and *Vibrio owensii*. After cloning and expression, the molecular weight (Mw) of purified Mg9373 was quite different from typical β-glucosidases previously reported. Generally, Mw of most β-glucosidases varied from 30 to 100 kDa [46], for example, BglB (50 kDa) from the gut microbiota of the termite *Reticulitermes santonensis* [47], BglNH (51 kDa) from *Streptomyces* sp. of deep sea sediment [48], BglD5 (52 kDa) from *Jeotgalibacillus malaysiensis* [49], TaBgl2 (52 kDa) from *Trichoderma asperellum* isolated from rotten oil palm fruit [50], BGL0224 (55 kDa) from *Oenococcus oeni* isolated from acidic tomato broth (ATB) [46], BglY (81 kDa) from *Paenibacillus* sp strain C7 of Bear Meadows Natural Area [51], and *Mt*bgl3a (90 kDa) from *Myceliophthora thermophila* [52]. 

### 3.4. Biochemical Characterization of Mg9373

After purification, we evaluated the effect of pH and temperature on the enzymatic activity of Mg9373. The acidic stability drops rapidly below pH 4.0 (Figure 6B), which is relatively low in comparison to the acidic stability of BGL0224, where 50% of its activity was retained at pH 6.0 [46]. However, the stability of Mg9373 under basic conditions was quite high, with >35% activity within the pH range of 7.0–9.0. When the pH is increased to the range of 9.0–11.0, Mg9373 also showed a measurable level of enzyme activity. The alkali stability of Mg9373 observed here is higher than that of BGL0224 [46]. The relative activity of Mg9373 remained at a high level (>40%) when the temperature was between 10 °C and 45 °C (Figure 6B), indicating that the enzyme is a mesophilic enzyme. The optimal catalytic temperature of reported β-glucosidase enzymes from microorganism is above 40 °C, such as β-glucosidase BGL0224 with an optimal catalytic temperature of 50 °C [46]. In addition, the optimal catalytic temperature of β-glucosidases from plant sources is approximately 65 °C, such as β-glucosidase isolated from legumes *Dalbrgia cochinchinensis* and *Dalberga nigrescens* [53]. In general, the optimal catalytic temperature of β-glucosidases from animals, plants, and microorganism were higher than that isolated from coral microorganism, including Mg9373. For example, r-BglA49 [54] was cloned from symbiotic *Serratia* sp. TN49 of *B. horsfieldi* larvae, and revealed an optimal catalytic temperature and pH of 37 °C and 7.5, respectively. Most metal ions were found to enhance Mg9373 activity. Interestingly, activity increased more than three-fold in the presence of 10 mM Mn^2+^, and 1.4-fold in the presence of 10 mM of Zn^2+^; however, it has been reported that 10 mM Zn^2+^ and Mn^2+^ inhibited the activity of other β-glucosidases, such as r-BglNH [48]. In addition, a two-fold increase in activity was observed in the presence of Co^2+^, which is different from other β-glucosidases, of which the activity generally increases by nearly 80% under similar conditions [48,52]. The r-BglNH is a novel salt-tolerant and glucose-enhanced β-glucosidases cloned from marine *Streptomycete*, which exhibited good enzyme activity under conditions of high salinity and high glucose [48]. In the presence of 5 M NaCl, the relative activity of Mg9373 was more than 20% of the control; however, the relative activity of the r-BglNH was only 10%. Similarly, in the presence of 10% glucose, the relative activity of Mg9373 was more than 65%, but that of r-BglNH was only 60%. These differences may be attributed to multiple environmental factors of coral reefs which lead to symbiotic bacteria gain novel genetic flexibility and adaptability. Coral growth and health are known to be strongly linked to temperature and salinity, which significantly affect the microbial community structure and function [55]. Mg9373 displayed relative high activity at the optimum temperature and salinity for coral growth around 26–27 °C and ~35%, indicating it has good micro-environmental adaptability in coral holobiont. This indirectly reflects the diversity and specificity of β-glucosidase genes in coral symbiotic bacteria. Overall, these results suggest that there may be abundance of novel β-glucosidase enzymes with high activity and tolerance in coral symbiotic microorganisms that have a great potential for applications.

## 4. Materials and Methods

### 4.1. Coral Sample Collection 

With the field permit by the Sanya natural reserve management office, coral samples were collected using a hammer and chisel at a depth of 5–10 m from Luhuitou Coral Reef, located in the southern part of Hainan Island, to the east of Sanya Bay, and west of the Luhuitou Peninsula in China (109°28′ E, 18°13′ N). All coral samples were divided into bracket coral, massive coral, and branching coral, by appearance, morphology, and bone. Coral samples were divided into 2–3 cm pieces, organized by species, and stored in sealed bags in an ice incubator. 

### 4.2. Isolation and Purification of β-Glucosidase-Producing Bacteria

All coral samples were identified through ecological and morphological characteristics, and the 2–3 cm pieces were diluted in 10 mL of sterile seawater and homogenized by vortexing with sterile 3 mm glass beads for 10 min at speed setting of 6.0 (Vortex-Genie, Bohemia, NY, USA). A portion of the resulting homogenates were removed as impurities after a short centrifugation period, and the rest homogenates were centrifuged through a gradient consisting of 10, 20, 30, 40, and 50% sucrose at 240× *g* for 20 min [56]. The resulting six sucrose layers were diluted 1000 times with sterile seawater. Aliquots of 0.1 mL of the diluted suspensions were plated on DMA media with three replicates (1/10 MA, MA: 0.5% peptone *W/V*, 0.1% yeast extract, 0.01% ferric citrate, 0.05% glucose, 1.5% agar powder, 500 mL distilled water, and 500 mL sea water) and incubated at 25 °C, until colonies appeared [57]. Colonies with different morphological characteristics were selected and streaked on the solid medium until uniform colonies were observed [58]. Subsequently, pure strains were transferred to screening plates (DMA solid plates supplemented with 0.05% esculin) and incubated at 25 °C. Isolated β-glucosidase-producing colonies, which appeared brown and included a dark zone surrounding the colony border, were incubated in DMA without agar, at 28 °C. The resulting purified bacterial solutions were stored in 20% glycerol at −80 °C.

### 4.3. Molecular Identification of the Bacterial Strains 

After incubation in the liquid screening medium without agar, the genomic DNA of cultivable β-glucosidase-producing bacteria was extracted using the FastDNA® Spin Kit for Soil, according to the manufacturer’s instructions (MPBIO, Irvine, CA, USA). Extracted 16S rRNA fragments were amplified using the universal primer pair 27F (5’-AGAGTTTGATCCTGGCTCAG-3’) and 1492R (5’-TACGGCTACCTTGTTACGACTT-3’). The PCR reaction was carried out at 95 °C for 4 min, followed by 30 cycles of 94 °C for 45 s, 54 °C for 45 s, 72 °C for 120 s, and finally 72 °C for 10 min. The purified PCR product was sequenced by Sangon Biotech (Shanghai, China). Bacterial strains were identified by comparison with available 16S rRNA gene sequences in the EZBioCloud (https://www.ezbiocloud.net) and the NCBI database (https://blast.ncbi.nlm.nih.gov) using a BLAST approach, to determine their approximate phylogenetic affiliation and closest relatives. Isolate with the same or only one base difference of 16S rRNA gene sequence were categorized as the same strain. A phylogenetic gene tree was generated from 16S rRNA using the neighbor-joining method with MEGA package version 5.0. The 16S rRNA gene sequences of all β-glucosidase-producing bacteria and full-length gene sequences encoding novel β-glucosidases in the study were deposited in GenBank database under accession numbers MW320536-MW32019 and MW362217-MW362219, respectively. 

### 4.4. Diversity of the β-Glucosidase Gene

A portion of genomic DNA was sequenced by Majorbio Bio-Pharm Technology Co., Ltd. (Shanghai, China) using next-generation sequencing platforms. All β-glucosidase nucleotide sequences were annotated and selected. Then, 68 sequences of GH1 and 84 sequences of GH3 family translated using the ExPASy tool (http://www.expasy.ch/tools). All deduced amino-acid sequences were prepared using the ClustalX, and DNAMAN (Version 6.0, LynnonBiosoft, San Ramon, CA, USA), and redundancies were removed with CD-HIT, resulting in a sequence identity of 95% [59]. Phylogenetic analysis and the preparation of a phylogenetic tree were completed using MEGA version 5.0 software and neighbor-joining (NJ) method.

### 4.5. Cloning and Expression of the β-Glucosidase Gene

Gene protein and gene ontology annotation were carried out with the use of the NCBI database. The open reading frame of the β-glucosidase gene was analyzed using the NCBI ORF finder (https://www.ncbi.nlm.nih.gov/orffinder/). A novel β-glucosidase gene, named Mg9373, was chosen for full-length gene cloning using the following primer pair: F_9373_ (5’-AACGTGCTACGAACAAAGAAAACAA-3’) and R_9373_ (5’-GATTGTGGTCGAACACTTCTTAGCG-3’) (GenBank Accession No. MT 795183). The PCR conditions were as follows: one cycle of 95 °C for 90 s; 30 cycles of 95 °C for 30 s, 62 °C for 30 s, and 72 °C for 1.5 min, followed by a final extension period of 72 °C for 10 min. The PCR product was purified using Magen Hipure Gel Pure DNA^®^ Mini Kit, (Magen, Shanghai, China) and ligated into the pEASY-E1(+) expression vector (TransGen Biotech, Beijing, China). The resulting recombinant plasmid containing the Mg9373 gene was transformed into *Escherichia coli* DH5a cells (TransGen Biotech, Beijing, China) using the heat shock method. Purified recombinant plasmid was subsequently transformed into *E. coli* BL21 (DE3) (TransGen Biotech, Beijing, China). Recombinant cells harboring pET-mg9373 were incubated in LB medium containing ampicillin (100 mg·mL^−1^), shaken at 180 rpm and at 37 °C. Once the bacterial culture reached an OD_600_ of 0.4–0.6, IPTG was added to a final concentration 0.6 mM for induction of protein expression at 22 °C and 180 rpm for 20 h.

### 4.6. Purification and Identification of Mg9373

Induced *E. coli* BL21 (DE3) cultures were harvested and washed twice with PBS buffer (pH 7.4), and lysed via sonication for 20 min in an ice-water mixture. The lysate was centrifuged at 8000× *g* for 20 min at 4 °C. The centrifuged lysate was applied to a Ni^2+^-NTA agarose gel column for purification. The recombinant protein was eluted with column buffer (20 mM Tris-HCl, pH 8.0, 300 mM NaCl, and 200 mM imidazole) and then dialyzed three times in deionized double-distilled H_2_O at 4 °C. The purified enzyme was analyzed with sodium dodecyl sulfate-polyacrylamide gel electrophoresis (SDS-PAGE). The protein concentration was determined by the Bradford method with bovine serum albumin as standard [60].

### 4.7. Effect of pH, Temperature, and Metal Ions on the Activity and Stability of Mg9373

For the evaluation of enzyme activity, *p*-nitrophenyl-β-D-glucopyranoside (*p*NPG) (Beijing Solarbio Science & Technology, Beijing, China) was used as the substrate for Mg9373. The optimum pH was determined by assaying with a variety of buffers at different pH values (pH 3.0–11.0). The buffers used were 0.2 M McIlvaine buffer (pH 3.0–8.0), and 0.05 M glycine-NaOH buffer (8.0–11.0). To determine pH stability, purified Mg9373 was preincubated in the above-described buffers for 1 h on ice. After preincubation, 2.5 mM *p*NPG (10 μL) was added to the mixture, followed by incubated at 35 °C for 5 min, and the addition of 1 M Na_2_CO_3_ (100 µL) to terminate the reaction. The optimum temperature was determined within the range of 0 °C to 70 °C, in phosphate buffer saline (PBS, pH 7). The thermostability of Mg9373 was determined by measuring the residual activity after incubating the enzyme at a given temperature for 1 h in PBS, followed by incubation on ice for 10 min, the addition of 25 mM substrate (10 µL) at the optimum temperature for 30 min, and finally the addition of 1 M Na_2_CO_3_ (100 µL) to terminate the reaction. To investigate the effects of different metal ions on enzyme activity, purified Mg9373 was preincubated with 10- and 20-mM concentrations of metal ions, including Mn^2+^, K^+^, Mg^2+^, Hg^2+^, Zn^2+^, Ca^2+^, Cu^2+^, Ni^2+^, Co^2+^, and EDTA. The purified recombinant enzyme (10 µL) was incubated with the metal ions for 1 h in the optimum pH buffer on ice, then 25 mM substrate (10 µL) was added at the optimum temperature for 5 min, and finally, the reaction was terminated with 100 µL 1 M Na_2_CO_3_. The effect of organic solvents on β-glucosidase activity was studied by incubating the enzyme with different solvents, including DMSO, methanol, acetone, ethanol, acetonitrile, isopropyl alcohol, butanol, isoamyl alcohol, benzene, toluene, and hexane with final concentrations of 10% and 20% (*v*/*v*). Purified enzyme was incubated for 1 h at the optimum temperature and pH with the various solvents, then 25 mM substrate (10 µL) was added at the optimum temperature for 5 min, and finally, the reaction was terminated with 100 µL 1 M Na_2_CO_3_. To determine the effect of salinity on enzyme stability, Mg9373 was incubated with different concentrations of NaCl (0.5, 1, 1.5, 2, 2.5, 3, 4, and 5 M) on ice for 30 min in optimum pH buffer, then 25 mM substrate (10 µL) was added at the optimum temperature for 5 min, and finally, the reaction was terminated with 100 µL 1 M Na_2_CO_3_. To determine the effect of glucose and ethanol on stability, Mg9373 was added to 80 µL of citrate buffer (pH 6.0, 50 mM) and incubated with various concentrations of glucose (5–20% (*M*/*v*)) and ethanol (5–30% (*v*/*v*)) on ice for 30 min in optimum pH buffer, then 25 mM substrate was added to the mixture at the optimum temperature for 30 min, and finally, the reaction was terminated with 100 µL 1 M Na_2_CO_3_.

### 4.8. Enzyme Activity and Kinetic Assay of Mg9373

Mg9373 activity was determined in a volume of 100 µL that included 10 µL of appropriately diluted enzyme, 10 µL of substrate (2.5 mM final concentration *p*NPG), and 80 µL of PBS buffer (pH 7.0). After incubation at 35 °C for 5 min, the reaction was terminated with 100 µL of 1 M Na_2_CO_3_. The amount of liberated *p*NPG was measured by detecting the absorption of the reaction mixture at 405 nm. The amount of enzyme was indicated as one unit of β-glucosidase activity (U) that hydrolyzed 1 µmol *p*NPG per min under optimal conditions [61]. The kinetic parameters of Mg9373, including *K*_m_ and *V*_max_, were determined by measuring the rate of hydrolysis of *p*NPG at various concentrations of the enzyme in PBS buffer (pH 7.0) at the optimum temperature for 5 min. The hydrolysis of *p*NPG by Mg9373 was investigated using Michaelis-Menten plots.

## Figures and Tables

**Table 1:** Cumulative list of cultivable β-glucosidase producing bacteria in corals.

CORAL GENERA								Total and Rate (%)
		*Pocillopora*	*Acropora*	*Porites*	*Favia*	*Turbinaria*	*Montipora*	
**GENERA DISTRIBUTION**								
**Fimicutes (6)**	*Bacillus*		1	1	3			5 (5.75%)
	*Lysinibacillus*						1	1 (1.15%)
	*Brevibacterium*		1					1 (1.15%)
	*Cytobacillus*			1			2	3 (3.45%)
	*Fictibacillus*				3			3 (3.45%)
	*Exiguobacterium*	2		2	2		3	9 (10.35%)
**Proteobacteria (11)**								
	*Photobacterium*				14			14 (16.09%)
	*Sphingobium*	3			1	5		9 (10.35%)
	*Brevundimonas*	1			1			2 (2.30%)
	*Paracoccus*	9		3				12 (13.79%)
	*Celeribacter*			1				1 (1.15%)
	*Pseudovibrio*		1		1			2 (2.30%)
	*Stenotrophomonas*						1	1 (1.15%)
	*Pseudomonas*					3		3 (3.45%)
	*Pseudoalteromonas*		1					1 (1.15%)
	*Alteromonas*		2				1	3 (3.45%)
	*Vibrio*	1	1	6	1		2	11 (12.64%)
**Actinobacteria (4)**								
	*Microbacterium*			2		1		3 (3.45%)
	*Rothia*		1					1 (1.15%)
	*Nocardia*					1		1 (1.15%)
	*Brachybacterium*		1					1 (1.15%)
**Total strain number (87)**		15	9	16	26	10	10	87

*Pocillopora*, *Acropora*, *Porites*, *Favia*, *Turbinaria*, and *Montipora* were represented by A, B, C, D, E, and F.

**Table 2:** Compared with cellulase and hemicellulose from coral and *Holotrichia parallela.*

Family	Cazy-Activities	Coral Gene	*Holotrichia parallela* Gene
GH1	Glucosidase	68	ND
GH2	Galactosidase	27	24
GH3	Glucosidase	84	0
GH5	Endoglucanase	14	0
GH8	Endoglucanase	11	27
GH11	Xylanase	1	14
GH16	Glucanase	28	ND
GH39	Xylosidase	5	0
GH52	Xylosidase	1	0

Comparison of hydrolase family genes from coral symbiotic microbes and terrestrial herbivorous insect Hindgut microbes. Number of genes in numbers. ND means not detected.

**Table 3:** Effect of 10 mM and 20 mM metal ions and chemical reagents on the MG9373 activity.

Substance	Relative Activity (%) ^a^	
	10 mM	20 mM
Control	100 ^b^	100
Ni^+^	65.4 ± 7.2	115 ± 26.9
Zn^2+^	140 ± 23.2	76 ± 1.2
Cu^2+^	43 ± 0.4	34 ± 4.6
Mn^2+^	351 ± 19.4	272 ± 39.5
Mg^2+^	108 ± 15.1	115 ± 14.8
Co^2+^	222 ± 18.8	ND
Hg^2+^	13 ± 0.8	13 ± 1.4
Ca^2+^	246 ± 9.3	261 ± 13.9
K^+^	74 ± 5	126 ± 10.0
EDTA	154 ± 9.8	218 ± 32.3

^a^ Assay was performed under optimum conditions; ^b^ Values represent the means ± SD (*n* = 3). ±, standard error; ND, not detected.

**Table 4:** Effect of various 10 or 20% organic solvents on MG9373 activity.

Solvent		Log P_OW_	Relative Activity (%) ^a^	
			10% (*v/v*)	20% (*v/v*)
Control		--	100 ^b^	100
DMSO		−1.35	47 ± 2.2	19 ± 0.9
Methanol		−0.76	9 ± 1.6	10 ± 0.5
Acetone		−0.24	30 ± 2.2	15 ± 2.4
Ethanol		−0.24	57 ± 8.8	29 ± 3.4
Acetonitrile		−0.34	15 ± 3.3	19 ± 3.5
Isopropyl	alcohol	0.16	44 ± 6.3	24 ± 4.9
Butanol		0.8	12 ± 1.4	15 ± 0.3
Isoamyl	alcohol	1.28	31 ± 4.6	54 ± 16.9
Benzene		2.13	79 ± 3.1	148 ± 30.2
Toluene		2.4	88 ± 12	153 ± 69.0
Hexane		3.5	115 ± 14.1	216 ± 20.2

^a^ Assay was performed under optimum conditions. ^b^ Values represent the means ± SD (*n* = 3) relative to the untreated control samples.

## Data Availability

Not applicable.

## Abbreviations

IPTG	isopropylthio-β-galactoside
SDS-PAGE	sodium dodecyl sulfate polyacrylamide gel electrophoresis
*p*NPG	*p*NP-β-D-glucopyranoside
